# The changing epidemiology of hepatitis B and C infections in Nanoro, rural Burkina Faso: a random sampling survey

**DOI:** 10.1186/s12879-019-4731-7

**Published:** 2020-01-15

**Authors:** Moussa Lingani, Tomoyuki Akita, Serge Ouoba, Shintaro Nagashima, Palwende Romuald Boua, Kazuaki Takahashi, Basile Kam, Aya Sugiyama, Théodore Nikiema, Chikako Yamamoto, Athanase Somé, Karim Derra, Ko Ko, Hermann Sorgho, Zekiba Tarnagda, Halidou Tinto, Junko Tanaka

**Affiliations:** 1 Department of Epidemiology, Infectious Disease Control and Prevention, Hiroshima University Graduate School of Biomedical and Health Sciences, 1-2-3 Kasumi, Minami-ku, Hiroshima, 734-8551 Japan; 2 Institut de Recherche en Sciences de la Santé, Direction Régionale du Centre Ouest (IRSS/DRCO), Nanoro, 11 BP 218 Burkina Faso; 3 Unité de Recherche Clinique de Nanoro (URCN), Nanoro, 11 BP 218 Burkina Faso; 4 Institut de Recherche en Sciences de la Santé, Bobo-Dioulasso, Burkina Faso

**Keywords:** Burkina Faso, Hepatitis B virus, Hepatitis C virus, Genotype, Vaccination

## Abstract

**Background:**

This study sought to provide up-to-date hepatitis B (HBV) and C (HCV) seroprevalence in rural Burkina Faso decade after hepatitis B vaccine was introduced in the national immunization scheduled for children.

**Methods:**

In 2018, a community-based, random sampling strategy with probability proportional to population size was conducted in Nanoro to investigate the prevalence of viral hepatitis in children and their mothers. Sociodemographic, vaccination history and risk factors were assessed by interview and health books. HBsAg rapid tests were done by finger prick and Dried Blood Spots (DBS) were collected for hepatitis seromarkers by chemiluminescence enzyme immunoassay. Positive samples underwent confirmatory PCR and phylogenetic analysis.

**Results:**

Data were presented on 240 mother-child pairs. HBsAg Prevalence was 0.8% in children and 6.3% in mothers. Hepatitis B core antibody positivity was 89.2% in mothers, 59.2% in children and was associated with age, sex and scarification. Hepatitis B surface antibodies prevalence was 37.5% in children and 5.8% in mothers. Good vaccination coverage was limited by home delivery. Phylogenetic analysis of HBV strains based on full genome sequences (*n* = 7) and s-fragment sequences (*n* = 6) revealed genotype A, E, and recombinant A3/E. Viral genome homology was reported in one mother-child pair. Anti-HCV prevalence was 5.4% in mothers, 2.1% in children and strains belonged to genotype 2.

**Conclusions:**

In Nanoro, HBsAg prevalence was low in children, intermediate in mothers and mother-to-child transmission persists. Home delivery was a limiting factor of Hepatitis B vaccination coverage. HBV genotype E was predominant and genotype A3/E is reported for the first time in Burkina Faso.

## Background

Although the global burden of viral hepatitis is gradually decreasing worldwide, it still represents an international health challenge particularly in low-and-middle income countries [1]. It is estimated that globally 1.34 million people died from viral hepatitis complications in 2015 [2]. Sub-Saharan African countries carry a heavy burden with over 60 million people living with hepatitis B (HBV) among whom, 2 million live in Burkina Faso [3, 4]. According to recent estimates, hepatitis B causes 54% of all liver cancer cases worldwide [5–7]. Meanwhile, hepatitis C virus (HCV) causes 10 to 25% of all liver cancers worldwide and affects 10–30 million people in West Africa [7–9]. Despite the introduction of hepatitis B vaccine into Burkina Faso’s national expanded program on immunization (EPI) for children in 2006, related death has increased and cost 2600 lives in 2015 [10]. The picture could even be worse given that accurate identification of death causes in sub-Saharan Africa is still a challenge [11]. In African regions, transmission is reported to be mainly perinatal through mother to child transmission or horizontal transmission through exposure to infected body fluids [12, 13].

Elimination of hepatitis B infection can achieve in a particular country when national government started to develop and implement efficacious strategies including free children vaccination against HBV, case detection and treatment with effective drugs [12, 13]. Elimination of HCV is also envisioned since highly effective direct antiviral agents (DAA) are now available in low-income countries and are reported to cure infection within 10 to 12 weeks of treatment [14, 15].

Since 2006, universal vaccination against HBV was started as part of Expanded Programme of Immunization (EPI) but Hepatitis B birth dose is not recommended to all new born till now. Specific to HBsAg positive mother, new guideline was recently released in September, 2019 in which all HBsAg positive mothers except those with HBV DNA < 2000 IU/ml should be treated with Tenofovir (TDF) and the birth dose should be given to the baby. Burkina Faso developed a 5-years strategic plan in 2017 to guide efforts based on improving vaccine coverage in infants, supply of effective antiviral drugs, and emphasizing on raising awareness. However, detail epidemiological data to support these efforts is still limited as well as evidence of the impact of current interventions [10, 16]. A severe limitation in this respect is the scarcity of reliable prevalence data from population-based studies. A recent meta-analysis reported an overall HBV prevalence of 9.4% in the country with however important regional variations and large estimation intervals [3]. These variations could be related to the characteristics of the diagnostic tests used and the specific populations screened like blood donors, pregnant women or HIV infected people. A recently published study reported a nationwide prevalence of 7.8 and 3.2% respectively for HBV and HCV among women with data collected since 2010 in HIV negative adult population [16]. To this regard, more recent community-level data is still needed to evaluate the impact of interventions in the rural area and subsequently develop strategies for improvement. Using HemaSpot™ (Spot on Sciences, USA) blood collection device, we investigate HBV and HCV infections seroprevalence and risk factors. Genotypic characterization has been performed, and key risk factors among children and their mothers living in Nanoro, at top north of centre west region of Burkina Faso were investigated. Our population-based study is expected to provide updated and additional information for adequate planning of preventive measures against viral hepatitis infections.

## Methods

### Study design, site and participants

This was a cross-sectional survey conducted among children born after 2006 and their respective mothers in Nanoro health and demographic surveillance system area (HDSS), Centre-west region of Burkina Faso. The study site covers an updated population size of 63,000 inhabitants. The study area was described elsewhere [17]. Nanoro is a rural area located in the Centre-West of the country. In this area, health care is provided by seven peripheral health posts and one referral hospital. Based on an anticipated hepatitis B surface antigen (HBsAg) prevalence in the rural area of 17.3% in Burkina Faso [3], confidence level of 95%, precision of 5, and 10% maximum missing data, the minimum sample size for mothers is 240 participants. As for children required sample size is less than 240, because the prevalence among children is thought as lower than that among adults. Therefore, the required sample size was totally 240 pairs of children and their mothers. The study on HBsAg prevalence in rural area of Burkina Faso is limited. The study area was randomly selected among all rural area of Burkina Faso. This study population covers 0.46% of all rural population in Burkina Faso. All 24 villages of the Nanoro HDSS were selected. We conducted sampling procedure as follows: First mothers were selected by random sampling with probability proportional to village size. Second, children were selected using simple random sampling from children of selected mothers when mother have more than one eligible child. Then, a list of 240 pairs of mothers above 15 years old and their child was generated from the HDSS database. Selected potential participants were visited at home by study investigators. Informed consent was obtained before study related activities were performed. If the woman has more than an eligible child aged one to 11 years old, we selected the participant (child) among eligible children using an automatic random number generator (RNG).

### Questionnaire survey

Trained study investigators administered a standardized questionnaire to all study participants (Additional file 1: Appendix 1a and 1b). Socio-demographic data such as age, sex, ethnic group, educational level, occupation, marital status, vaccination history; and potential risk factors including surgery, blood transfusion, injections, tattoos, skin-piercings, scarification, birthplace, were collected. The vaccination history was taken mainly from vaccination card. If the child has no vaccination card, the information was obtained by the recall-memory of their mothers. For the mother, the information could only be obtained by their recall memory.

### Serological and molecular assays

A rapid point of care Alere Determine™ HBsAg test strips, sensitivity 95–100% and specificity 96–100% (Abbott Japan Co., Ltd.) using 1 drop (20 μl) was performed using blood obtained from participants finger pricks. Completely vaccinated referred to children who received 3 doses of pentavalent vaccine. Three drops of blood (60 μl) were collected on HemaSpot™ sampling devices, dried and stored at minus 80 °C in Nanoro and shipped to Hiroshima university for laboratory analysis. The HemaSpot™ comprises absorbent filter paper separated into eight fragments in a fan-shape with a central hole. Three fragments were detached and eluted at room temperature in 600 μL of elution buffer [Tris-buffered saline (TBS): 50 mM Tris, 150 mM NaCl, 0.1% Proclin 300 and 0.05% Tween 20 at pH 7.2]. The mixtures were stirred in shaking plates for 1 hour and subsequently centrifuged at 12000 rpm for 5 min at 4 °C and the supernatants were separated. Using Lumipulse G1200 (Fujirebio Inc. Inc., Japan), a chemiluminescent enzyme immunoassay (CLEIA) was used to detect HBsAg (Lumipulse® -II HBsAg, Fujirebio Inc., Japan with reported sensitivity of 100% and specificity of 99.7% [18]) with a cut-off index (COI) of 1.0, HBcAb (Lumipulse® HBcAb-N, Fujirebio Inc., Japan, with reported sensitivity of 88.4% and specificity of 95.2% [18]) COI of 0.6, HBsAb (Lumipulse® HBsAb-N, Fujirebio Inc., Japan, with reported sensitivity of 95% and specificity of 100% [18]) COI of 3.4, HBeAg (Lumipulse® -I HBeAg, Fujirebio Inc., Japan, with reported sensitivity of 97.6% and specificity of 98.2% [18]) COI of 1.0, HBeAb (Lumipulse® HBeAb-N Inc., Fujirebio, Japan, with reported sensitivity of 100% and specificity of 95.9% [18]) COI of 50.0. We considered the positivity of HBsAg or HBcAb sero-positivity as indicative of “HBV exposure”. Samples positive to HBsAb and negative for both HBsAg and HBcAb were classified as “serologically vaccinated”. Those with negative test results to HBsAg, HBsAb and HBcAb were designated as “susceptible” and at risk of HBV infection. To investigate HCV infection, HCV antibody (HCVAb) positivity (LumipulseII®Ortho- HCV, Ortho Clinical Diagnostics, Japan) with a cut-off value of 0.6 was measured using CLEIA.

### Molecular assay and genome sequencing

In HBsAg positive samples, confirmatory tests were performed to detected and quantify HBV DNA by nucleic acid amplification test (NAT). At first, nucleic acid was extracted from HemaSpot™ using SMITEST EX-R&D (Medical and Biological Laboratories co., LTD, MA, USA). To detect HBV DNA, nested PCR assays with Applied Biosystems MiniAmp Plus Thermal Cycler (Thermo Fisher Scientific, Tokyo, Japan) were performed using primers obtained from Minegishi K et al work [19]. For viral load assessment, nucleic acid extracts were amplified and measured using a real-time PCR TaqMan Fast Universal PCR Master Mix (2X); Applied Biosystems, StepOne™ (Thermo Fisher Scientific, Tokyo, Japan) with primers and probe from HBV s-region gene [20, 21]. Hepatis C virus RNA was also detected using nested PCR amplification with in-house designed primer set from the conserved core region gene.

Genome sequencing for genotype analysis was conducted. To this regard, PCR products were generated using PrimeStar GXL (Takara Bio Inc., Shiga, Japan). Subsequently, a direct BigDye Terminator v3.1 Cycle Sequencing Kit; (Applied Biosystems, Foster city, CA, USA) was used.

### Data management and statistical analysis

Study data were collected and managed using REDCap electronic data capture tools hosted at the Clinical Research Unit of Nanoro (CRUN). Data were analysed using Stata, Version 15 (StataCorp. 2017, TX). Proportions were estimated with 95% exact confidence intervals (95%CI). The χ2 or Fisher’s exact test were used as appropriate to compare proportions between groups. For risk factors of HBsAg positivity, HBV exposure and HBcAb positivity in children and mothers, crude odd-ratios (OR), adjusted OR and their 95% CIs were calculated by a univariate logistic regression and multivariate logistic-regression using a stepwise selection procedure (method of increasing and decreasing based on *p*-value threshold: both of probability to enter and probability to leave are 0.3) Covariate for analysis in children are age group, one pentavalent at least, completely vaccine, gender, ethnic, blood transfusion, birth assistance and traditional scarification. Covariate for analysis in mothers are age group, occupation, partner occupation, schooling status, birth place, pregnancy, genital mutilation, marital status, birth assistance, know about MTCT and Heard about hepatitis. *P*-value < 5% was considered statistically significant.

### Ethical considerations

The study protocol was approved by the ethics committee for epidemiological research of Hiroshima University (Certificate E-1257) and the ethics committee of the ministry of health in Burkina Faso (Deliberation CERS-2018-02-023). All participants signed an informed consent before they can enter the study. Participants tested positive to hepatitis infection were referred to a specialized centre.

## Results

### Demographic characteristics of study participants

A total of 480 respondents including 240 children (born between 2007 and 2016) and 240 mothers were included in the study. Mothers average age was 33.2 ± 7.8 years, with majority of them were farmers (93.3%) and 9.2% of them were pregnant. Children median age was 4.0 years (53.3% males) and 89.2% of them were born in healthcare facilities. Table 1 summarize the seromarkers positivity in relation to study population background’s characteristics.

**Table 1:** Serological markers positivity by DBS analysis in relation to the socio-demographic characteristics of study participants from Nanoro, Burkina Faso

		Total		HBsAg positive				HBsAb positive				HBcAb positive				Anti-HCV positive			
		*n*	%	*n*	%	(95%CI’)	*p*	*n*	%	(95%CI’)	*p*	*n*	%	(95%CI’)	*p*	*n*	%	(95%CI’)	*p*
Mother	Total	240	100.0	15	6.3	(3.5–10.1)	–	14	5.8	(3.2–9.6)	–	214	89.2	(84.5–92.8)	–	13	5.4	(2.9–9.1)	–
	Age group (years)																		
	[18–29]	80	33.3	7	8.7	(3.6–17.2)	0.580	2	2.5	(0.3–8.7)	0.228	71	88.7	(79.7–94.7)	0.407	3	3.7	(0.7–10.6)	0.616
	[30–39]	108	45.0	6	5.6	(2.1–11.7)		9	8.3	(3.9–15.2)		94	87.0	(79.2–92-7)		6	5.6	(2.0–11.7)	
	[40–60]	52	21.7	2	3.8	(0.4–13.2)		3	5.8	(1.2–15.9)		49	94.2	(84.0–98.8)		4	7.7	(2.1–18.5)	
	Educational status																		
	Unschooled	195	81.2	11	5.6	(2.8–9.8)	0.525	10	5.1	(2.5–9.2)	0.364	173	88.7	(83.4–92.8)	0.570	10	5.11	(2.5–9.2)	0.145
	Primary	23	9.6	2	8.7	(1.1–28.0)		2	8.7	(1.1–28.4)		22	95.6	(78.0–99.8)		3	3.0	(2.8–33.6)	
	Secondary	22	9.2	2	9.1	(1.1–29.6)		2	9.1	(1.1–29.1)		19	86.4	(65.1–97.1)		0	0.0	(0.0–15.4) ^b^	
	Ethnic group																		
	Mossi	223	92.9	14	6.3	(3.5–10.3)	0.679	13	5.8	(3.1–9.7)	0.653	198	88.8	(83.9–92.6)	0.384	10	4.5	(2.2–8.1)	0.045^a^
	Gourounsi	12	5.0	1	8.3	(0.2–38.5)		1	8.3	(0.2–38.4)		12	100.0	(73.5–100.0) ^b^		3	25.0	(5.5–57.2)	
	Others	5	2.1	0	0.0	(0.0–52.2) ^b^		0	0.0	(0.0–52.2) ^b^		4	80.0	(28.3–99.5)		0	0.0	(0.0–52.2) ^b^	
	Birth place																		
	Health center	79	32.9	5	6.3	(2.1–14.1)	0.942	5	6.3	(2.1–14.1)	0.313	68	86.1	(76.4–92.8)	0.274	5	6.3	(2.1–14.1)	0.934
	Home birth	105	43.8	6	5.7	(2.1–12.0)		8	7.6	(3.3–14.4)		93	88.6	(80.8–93.9)		5	4.8	(1.5–10.7)	
	Unknown	56	23.3	4	7.1	(1.9–17.3)		1	1.8	(0.0–9.5)		53	94.6	(85.1–98.8)		3	5.4	(1.1–14.8)	
	Pregnancy																		
	No	217	90.4	13	6.0	(3.2–10.0)	0.643	13	6.0	(3.2–10.0)	0.603	195	89.8	(85.0–93.5)	0.289	12	5.5	(2.9–9.4)	0.640
	Yes	23	9.6	2	8.7	(1.1–28.0)		1	4.3	(0.1–21.9)		19	82.6	(61.2–95.0)		1	4.3	(0.1–21.9)	
Child	Total	240	100.0	2	0.8	(0.1–3.0)	–	90	37.5	(31.3–43.9)	–	142	59.2	(52.6–65.4)	–	5	2.1	(0.7–4.8)	–
	Age group (years)																		
	[1–4]	134	55.8	1	0.7	(0.0–4.1)	0.689	53	39.5	(31.2–48.3)	0.460	68	50.7	(42.0–59.5)	0.003^a^	3	2.2	(0.5–6.4)	1.000
	[5–10]	106	44.2	1	0.9	(0.0–5.1)		37	34.9	(25.9–44.8)		74	69.8	(60.1–78.3)		2	1.9	(0,2–6.6)	
	Gender																		
	Female	112	46.7	2	1.8	(0.2–6.3)	0.217	35	31.2	(22.8–40.7)	0.061	73	65.2	(55.6–73.9)	0.076	1	0.9	(0.0–4.9)	0.375
	Male	128	53.3	0	0.0	(0.0–2.8) ^b^		55	42.9	(34.2–52.0)		69	53.9	(44.9–62.7)		4	3.1	(0.8–7.8)	
	Educational status,																		
	Preschool age	180	75.0	1	0.6	(0.0–3.1)	0.438	78	43.3	(36.0–50.9)	0.004^a^	98	54.4	(46.8–61.8)	0.036^a^	5	2.8	(0.9–6.3)	0.694
	Elementary	46	19.2	1	2.2	(0.0–11.5)		8	17.4	(7.8–31.4)		34	73.9	(58.8–85.7)		0	0.0	(0.0–7.7) ^b^	
	Unschooled	14	5.8	0	0.0	(0–23.1) ^b^		4	28.6	(8.4–58.1)		10	71.4	(41.9–91.6)		0	0.0	(0.0–23.1) ^b^	
	Ethnic group																		
	Mossi	224	93.3	2	0.9	(0.1–3.2)	1.000	85	37.9	(31.5–44.6)	0.770	131	58.5	(51.7–65.0)	0.208	5	2.2	(0.7–5.1)	1.000
	Gourounsi	11	4.6	0	0.0	(0–28.5) ^b^		3	27.3	(6.0–60.9)		9	81.8	(48.2–97.7)		0	0.0	(0.0–28.5) ^b^	
	others	5	2.1	0	0.0	(0.0–52.2) ^b^		2	40.0	(5.3–85.3)		2	40.0	(5.3–85.3)		0	0.0	(0.0–52.2) ^b^	
	Birth place																		
	Health center	214	89.2	1	0.5	(0.0–2.6)	0.205	85	39.7	(33.1–46.6)	0.042^a^	124	57.9	(51.0–64.6)	0.269	5	2.3	(0.7–5.3)	1.000
	Home birth	26	10.8	1	3.8	(0.1–19.6)		5	19.2	(6.5–39.3)		18	69.2	(48.2–85.6)		0	0.0	(0.0–13.2) ^b^	
	Completely vaccinated																		
	Yes	151	62.9	1	0.7	(0.0–3.7)	0.7041	61	40.4	(32.9–48.4)	0.227	80	53.0	(45.0–60.8)	0.011	4	2.6	(1.0–6.6)	0.4242
	No	89	37.1	1	1.1	(0.2–6.1)		29	32.6	(23.7–42.9)		62	69.7	(59.5–78.2)		1	1.1	(0.2–6.1)	
	Mother’s HBsAg																		
	Positive	15	6.3	1	6.7	(1.1–29.8)	0.010	5	33.3	(15.2–58.3)	0.731	8	53.5	(30.1–75.2)	0.6350	0	0.0	(0.0–24.6) ^b^	0.5596
	Negative	225	93.7	1	0.4	(0.0–2.5)		85	37.8	(31.7–44.3)		134	59.6	(53.0–65.8)		5	2.2	(1.0–5.1)	

*%* Percentage, *CI* Confident Interval, *HBsAg* Hepatitis B surface antigen, *HBsAb* Hepatitis B surface antibody, *HBcAb* Hepatitis B core antibody, *HBeAg* Hepatitis B envelop antigen; HBeAb, Hepatitis B envelop antibody; Anti-HCV, Hepatitis C antibody; *DBS* Dried blood spot; ^a^ Represents statistically significance, ^b^one-sided, 97.5% confidence interval

### Prevalence of hepatitis B infection based on the questionnaire survey

By rapid diagnosis test, HBsAg prevalence among children was 0.8% (2/240) and 5.8% (14/240) among mothers. One sample indeterminate by rapid diagnosis test, showed a positive result in the laboratory testing. The Cohen’s Kappa coefficient (κ) showed a very good degree of agreement between the rapid testing and the CLEIA method (0.97; 95%CI, 0.91–1.00).

From the questionnaire, 11.7% (28/240, 95% CI, 7.9–16.4) of mothers were aware of the existence of the hepatitis B vaccine. Sixty-three percent 63.0% (151/240, 95% CI, 56.4–69.0) of children received 3 doses of pentavalent vaccine. The proportion of completely vaccinated children was significantly higher in the under 5-year-old children than the older children (75.4% vs 47.2%, *p* < 0.001) while none of their mothers was completely vaccinated in this survey.

### Serological survey

#### Prevalence of HBV infection based on DBS

**Fig. 1:**
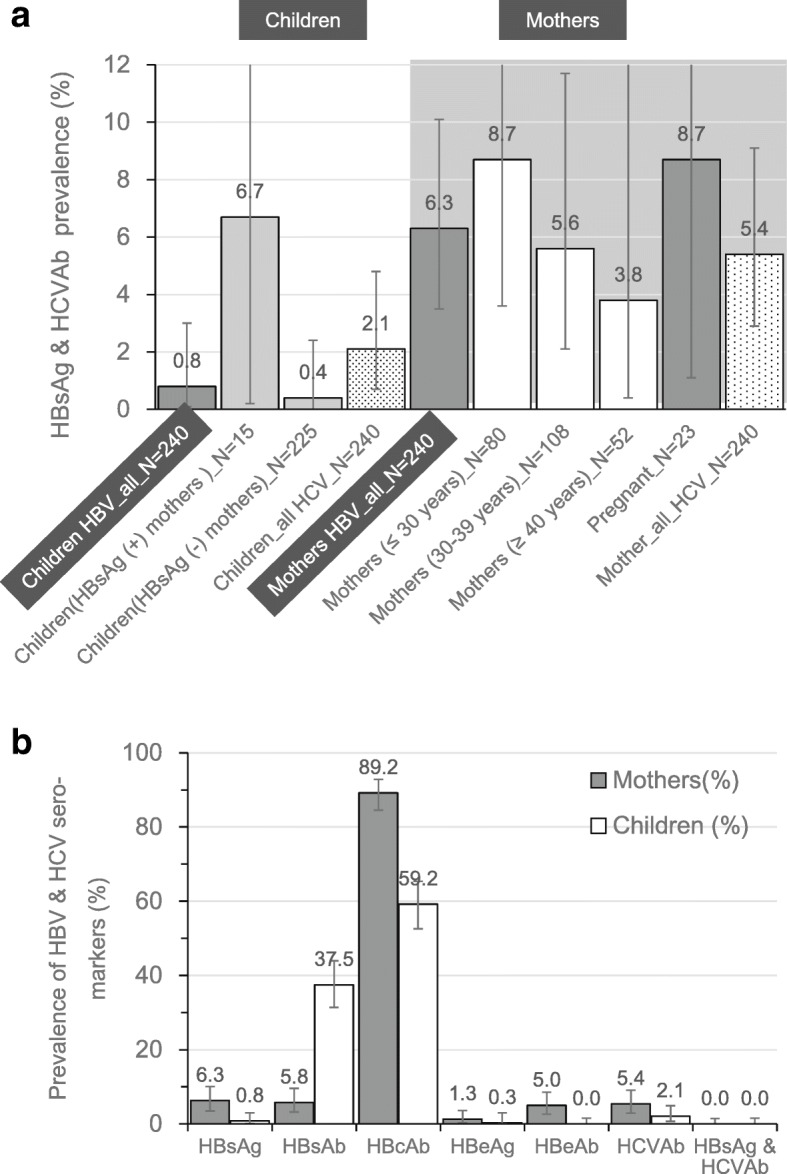
HBsAg and anti-HCV prevalence among mothers and children residing in Nanoro, rural area of Burkina Faso. **a** describes the extend of hepatitis B surface antigen and antibodies against hepatitis C virus among mothers and children assessed at the community level in Nanoro using dried blood spot samples collected on HemaSpot™. The white background presents data for the children and the dark background for the mothers. **b** describes the positive rates of each sero-marker of HBV (HBsAg, HBsAb, HBcAb, HBeAg, HBeAb) and HCV (HCVAb) for both mother and their children. The grey colour column represents the mothers and the while one represents the children

In Total, 6.3% (95% CI, 3.5–10.1) of mothers, 0.8% (95% CI, 0.1–3.0) of children, 8.7% (95% CI, 1.1–28.0) of pregnant women and 6.7% (95% CI, 0.2–31.2) of children born to a positive HBsAg mother were tested positive for HBsAg (Fig. 1a). Approximately 89.2% (95% CI, 84.5–92.8) of mothers and 59.2% (95% CI, 52.6–65.4) of children were classified as exposed (HBsAg / HBcAb positive) (Additional file 2: Appendix 2). Risk analysis showed significant higher risk of infection in over 5-year old children (aOR = 2.1, 95%CI, 1.2–3.8, *p* = 0.008), in female (aOR = 1.7, 95%CI, 1.1–2.9, *p* = 0.047) and in children with traditional scarification (aOR = 2.7, 95%CI, 1.1–6.7, *p* = 0.038) (Tables 2 and 3). High viral replication marker (HBeAg positive) was observed in 0.8% (95%CI, 0.1–3.0) of children and 1.3% (95%CI, 0.3–3.6) mothers (Fig.1b). In HBsAg carrier mothers, HBeAb, a surrogate for reduced viral replication, was positive in 60.0% (95% CI, 32.3–83.6%) of infected mothers while none of the children was positive (Fig. 1b).

**Table 2:** Univariate and multivariate analyses of risk factors of HBsAg sero-positivity, HBV exposure among mother participants in rural Nanoro, Burkina Faso, 2018

		n	%	HBsAg positivity (*n* = 15)							Exposure (HBsAg or HBcAb positive) ^a^ (*n* = 214)						
				Univariate analysis^b^				Multivariate analysis^c^			Univariate analysis^b^				Multivariate analysis^d^		
				n(+)	OR	(95%CI)	*p* value	aOR	(95%CI)	*p* value	n(+)	OR	(95%CI)	*p* value	aOR	(95%CI)	*p* value
Age group (years)	[18–29]	80	33.3	7	1.0						71	1.0			1.0		
	[29–39]	108	45.0	6	0.6	0.2–1.9	0.397	–	–	–	94	0.9	0.3–2.1	0.723	0.9	0.3–2.3	0.788
	[39–60]	52	21.7	2	0.4	0.1–2.1	0.288				49	2.1	0.5–8.0	0.293	2.1	0.5–8.9	0.304
Occupation	Farmer	224	93.3	13	1.0			–			201	1.0			–		
	others	16	6.7	2	2.3	0.5–11.3	0.298				13	0.5	0.1–1.9	0.300			
Partner occupation	Farmer	213	88.7	12	1.0			1.0			189	1.0					
	Civil servant	6	2.5	0	–			–			5	0.6	0.1–5.7	0.684	–		
	others	21	8.8	3	2.8	0.7–10.8	0.137	3.7	0.9–15.2	0.074	20	2.5	0.3–19.8	0.373			
Schooling status	Unschooled	195	81.3	11	1.0			–	–	–	173	1.0					
	Primary	23	9.6	2	1.6	0.3–7.7	0.561				22	2.8	3.6–21.8	0.326	2.7	0.4–21.7	0.333
	Secondary	22	9.2	2	1.7	0.3–8.1	0.522				19	0.8	0.2–2.9	0.743	0.8	0.2–3.2	0.791
Birth place	Health center	79	32.9	5	1.0			–	–		68	1.0					
	Home birth	105	43.8	6	0.9	0.3–3.1	0.861			–	93	1.3	0.5–3.0	0.613			
	Unknown	56	23.3	4	1.1	0.3–4.4	0.851				53	2.9	0.8–10.8	0.121			
Pregnancy	No	217	90.4	13	1.0			–	–	–	195	1.0			–		
	Yes	23	9.6	2	1.5	0.3–7.1	0.612				19	0.5	0.2–1.7	0.294			
Genital mutilation	No	140	58.3	11	1.0			1.0	0.1–1.3	0.133	124	1.0					
	Yes	100	41.7	4	0.5	0.2–1.6	0.232	0.4			90	1.2	0.5–2.7	0.726			
Marital status	Married	238	99.2	14	1.0			–			213	1.0			–		
	Unmarried	2	0.8	1	16.0	0.9–269.5	0.054				1	0.1	0.0–1.9	0.134			
Birth assistance	Health Skilled	79	32.9	5	1.0			–			68	1.0			–		
	Traditional skilled	83	34.6	5	0.9	0.3–3.4	0.936				74	1.3	0.5–3.4	0.552			
	Family member	14	5.8	0	–	–	–				12	1.0	0.2–4.9	0.971			
	other	64	26.7	5	1.3	0.3–4.5	0.730				60	2.4	0.7–8.0	0.146			
Know MTCT	No	192	80.0	9	1.0			–			170	1.0			–		
	Yes	48	20.0	6	2.9	1.0–8.6	0.054				44	1.4	0.5–4.3	0.535			
Heard about hepatitis	No	118	49.2	5	1.0			1.0			105	1.0			–		
	Yes	122	50.8	10	2.0	0.7–6.1	0.210	1.3			109	1.0	0.5–2.3	0.928			

In multivariate analysis, covariates were selected by stepwise procedure (method of increasing and decreasing based on p-value threshold: both of probability to enter and probability to leave are 0.3)

^a^ hepatitis B virus exposure (HBV): Sero-positivity to hepatitis B surface antigen (HBsAg) or hepatitis B core antibody (HBcAb), MTCT, mother to child transmission;

^b^ χ2– Test or Fisher Exact Test

^c^ Logistic regression analysis with the stepwise method: R^2^ = 0.0582, model *p*-value < 0.04, *n* = 240

^d^ Logistic regression analysis with the stepwise method: R^2^ = 0.0129, model *p*-value < 0.03, n = 240

OR, Odds Ratio, aOR, adjusted Odds Ratio, CI, Confident Interval

**Table 3:** Univariate and multivariate analyses of risk factors of HBsAg sero-positivity, HBV exposure among children participants in rural Nanoro, Burkina Faso, 2018

Child		n	%	Exposure (HBsAg or HBcAb positivity)^a^ (*n* = 142)							HBsAb positivity (*n* = 90)						
				Univariate analysis^b^				Multivariate analysis^C^			Univariate analysis^b^				Multivariate analysis ^d^		
				n(+)	OR	(95%CI)	p	aOR	(95%CI)	*p* value	n(+)	OR	(95%CI)	*p* value	aOR	(95%CI)	*p* value
Age group (years)	[1–4]	134	55.8	68	1.0		0.003^e^	1.0			53	1.0					
	[5–10]	106	44.2	74	2.2	1.3–3.8		2.1	1.2–3.8	0.008^e^	37	0.8	0.5–1.4	0.461			
One Penta at least	No	13	5.4	58	1.3	0.4–4.1		–			27	1.0			–	–	–
	Yes	227	94.6	84	1.0		0.451				63	2.1	0.6–7.7	0.279			
Completely vaccinated	No	89	37.1	62	2.0	1.2–3.5		1.7	0.9–3.1		29	1.0			–	–	–
	Yes	151	62.9	80	1.0		0.012^e^	1.0		0.076	61	1.4	0.8–2.4	0.228			
Gender	Female	112	46,7	73	1.7	1.0–2.7	0.077	1.7	1.1–2.9		35	1.0			1.0		
	Male	128	53.3	69	1.0			1.0		0.047^e^	55	1.7	1.0–2.8	0.062	1.6	0.9–2.7	0.104
Birth place	Health center	214	89.2	124	1.0			–			85	1.0			1.0		
	Home birth	26	10.8	18	1.6	0.7–3.9	0.273				5	0.4	0.1–0.9	0.048^e^	0.4	0.1–1.1	0.073
Ethnic group	Mossi	224	93.3	131	1.0			–			85	1.0					
	Gourounsi	11	4.6	9	3.2	0.7–15.1	0.143				3	0.6	0.2–2.4	0.479	–	–	–
	Other	5	2.1	2	0.5	0.1–2.9	0.418				2	1.1	0.2–6.7	0.925			
Blood transfusion	No	228	95.0	135	1.0			–			86	1.0			–		
	Yes	12	5.0	7	1.0	0.3–3.1	0.952				4	1.2	0.4–4.1	0.760		–	–
Birth assistance	Health Skilled	214	89.2	124	1.0			–			85	1.0			–	–	–
	Traditional skilled	12	5.0	10	3.6	0.8–17.0	0.101				2	0.3	0.1–1.4	0.130			
	other	14	5.8	8	1.0	0.3–2.9	0.953				3	0.4	0.1–1.5	0.185			
Traditional Scarification	No	211	87.9	120	1.0			1.0			81	1.0			–		
	Yes	29	12.1	22	2.4	1.0–5.8	0.057	2.7	1.1–6.7	0.038^e^	9	0.7	0.3–1.7	0.444		–	–

In multivariate analysis, covariates were selected by stepwise procedure (method of increasing and decreasing based on p-value threshold: both of probability to enter and probability to leave are 0.3)

^a^ hepatitis virus exposure: Seropositivity to hepatitis B surface antigen (HBsAg) or hepatitis B core antibody (HBcAb)

^b^ χ2– Test or Fisher Exact Test

^c^ Logistic regression analysis with the stepwise method: R^2^ = 0.0649, model *p*-value < 0.05, n = 240

^d^ Logistic regression analysis with the stepwise method: R^2^ = 0.0227, model *p*-value < 0.03, n = 240

^e^ Represents statistically significance

*OR* Odds ratio, *aOR* adjusted Odds ratio, *CI* Confident Interval

### Survey on vaccine effects

Among 240 mother-child pairs, only one mother had received hepatitis B vaccine for two times while 63.0% of children (151/240) had received complete 3 doses hepatitis B vaccine and 66.3% of children (159/240) had received at least 1 dose of hepatitis B vaccine. Approximately 5.8% of mothers and 37.5% of children had protective level of HBsAb (Additional file 2: Appendix 2). Among mothers, all were due to past infections while 60% of protected children were also secondary to past infections. Only 15.0% (95% CI, 10.7–20.1) of all children were immune by vaccination. This proportion of vaccine protected children was significantly higher in children born within health centres (*p* = 0.042). Even though this was not statistically significant in multivariate analysis, a higher trend of protected children was observed in children born in health facilities than those born at home (aOR = 0.4, 95%CI 0.1–1.1, *p* = 0.073).

### Hepatitis B surface antigen positive samples

**Fig. 2:**
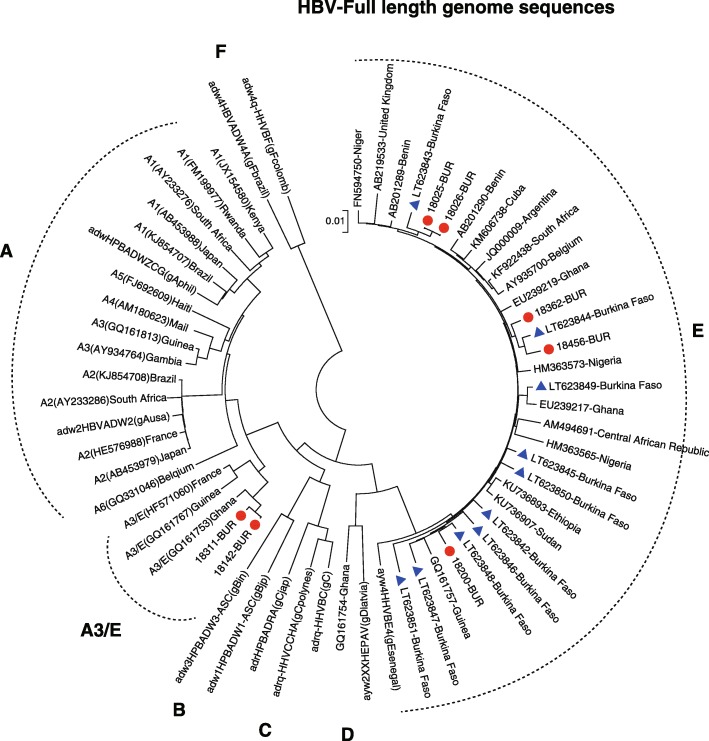
Evolutionary relationships of taxa of HBV The evolutionary history was inferred using the Neighbor-Joining method. The optimal tree with the sum of branch length = 0.83994352 is shown. The tree is drawn to scale, with branch lengths in the same units as those of the evolutionary distances used to infer the phylogenetic tree. The evolutionary distances were computed using the Maximum Composite Likelihood method and are in the units of the number of base substitutions per site. The analysis involved 62 nucleotide sequences. All positions containing gaps and missing data were eliminated. There were a total of 3054 positions in the final dataset. Evolutionary analyses were conducted in MEGA7.

Additional file 3: Appendix 3 summarises the characteristics of samples with positive HBsAg test results. HBV DNA was detected in 76.5% (13/17) including all positive children. The titter of HBV DNA was measured in 11 samples and viral load ranged from 5 copies/mL to 6.1E+ 07 copies/mL. Genotyping was successful in 13 samples, and HBV genotype E was predominant with 61.5% (8/13), followed by genotype A with 23.1% (3/13) and a recombinant genotype A/E 15.4% (2/13). The full length genome sequencing was completed in 7 samples. Case B18–025 (child) and case B18–026 (her mother) had 100% identical genetic sequences. After, phylogenetic analysis and comparison of the strain sequences with sequence in GenBank it was found that the reported genotype A/E was close to A3/E (GQ161753) Ghana and the genotype E strain 18–025-BUR, 18–200-BUR, and 18–456-BUR close to sequence identify in the western part of the country in Bobo-Dioulasso (Fig. 2) [22].

### Prevalence of HCV infection

**Fig. 3:**
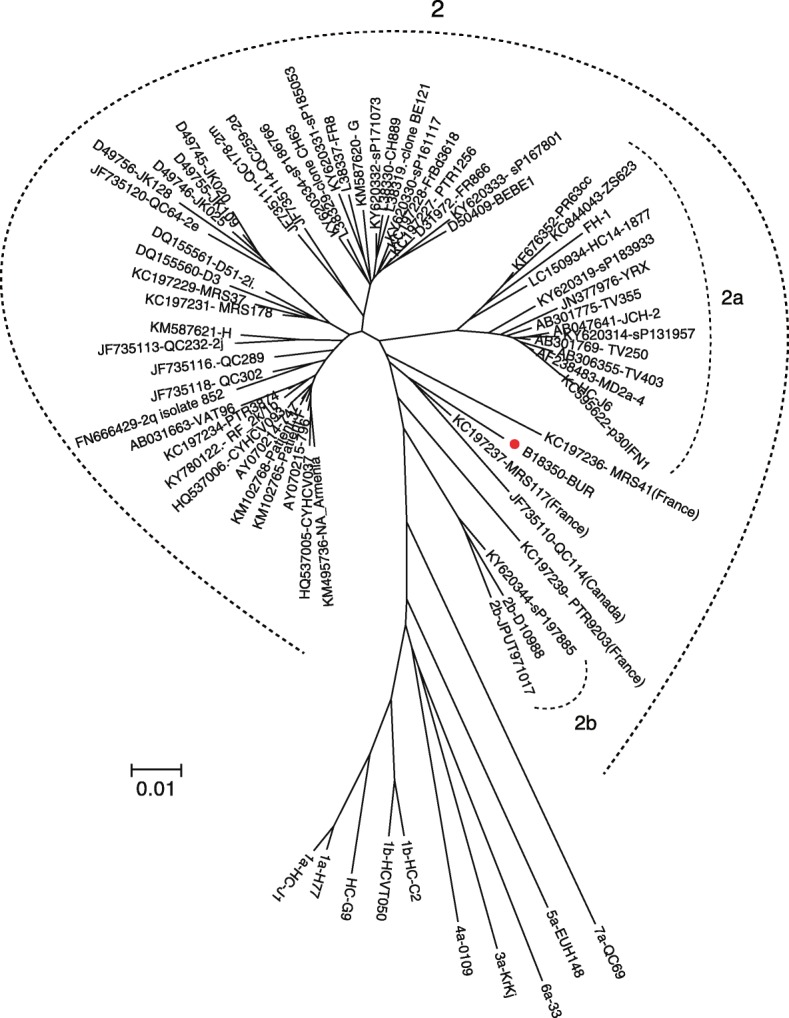
Evolutionary relationships of taxa of HCV. The evolutionary history was inferred using neighbor-joining method

HCV antibodies were positive among 5.4% (95% CI, 2.9–9.1) of mothers and 2.1% (95% CI, 0.7–4.8) of children. Virus RNA was tested and detected in one positive sample. Phylogenetic analysis reported hepatitis C genotype 2 (Fig. 3). Difference in anti-HCV rate was significant between the participant ethnic group in mothers (*p* = 0.045). By multivariate analysis in mother, history of blood transfusion yield OR of 6.2 (95%CI, 0.6–64.3) but it has no significant association to HCV infection (*p* = 0.125).

## Discussion

In this study, we investigated the prevalence and risks factors of hepatitis B and hepatitis C virus infections among children born after hepatitis B vaccine was introduced in the national EPI schedule and in their respective mothers. The study was conducted in Nanoro, a rural area of the centre-west region of Burkina Faso and a random sampling method was used to select a representative sample of women and their children. Burkina Faso is defined as a high transmission zone with HBsAg prevalence between 8.0–20.0%, HBcAb between 70.0–95.0% [22, 23]. Past studies have confirmed this assumption with HBsAg prevalence varying between 9.1 and 16.0% among adults with however data collected in the urban zone among specific populations including blood donors, pregnant women, HIV positive person, more likely to be at higher risk than the general population [3, 16, 24]. In this first study using a random sampling approach to assess all HBV infection seromarkers in rural Burkina Faso, a much lower HBsAg prevalence of 6.3% among adults mothers and 0.8% among children was reported. A higher prevalence among children (3.4%) was reported in the western region and was even higher in its rural area (7.1%) [25]. The lower HBsAg observed prevalence observe in the current study could be related to multiple interventions undertaken within the national viral hepatitis control program, among them the introduction of hepatitis B vaccine into the national EPI schedule as well as the hepatitis B vaccine clinical trials conducted in the area within the last decade. The impact of hepatitis B vaccine in reducing the circulating HBsAg was already reported [26]. However, vaccine completion rate was not satisfactory in children with 63.0% completely vaccinated during their childhood. This is quite alarming as vaccination is free for children. Therefore, it is urgent to improve awareness among population as only a small proportion of mothers (11.7%) in our study knew that vaccine could protect against hepatitis infection. The rate of exposure significantly increased with the older age, and this could be related to a larger vaccine coverage in younger children. Indeed, hepatitis B vaccine coverage was significantly higher in under 5 years age children than their elder peers (*p* < 0.001) and advocates for the improvement of HBV vaccine coverage in children. Pregnant women and children born to positive HBsAg carrier mothers were more at risk of infection and advocates the necessity to improve prevention in pregnant women and their new-born through either the administration of hepatitis B vaccination birth dose or by adequately diagnosing and treating high risk mothers during pregnancy to prevent transmission to their babies [27]. Indeed, pregnant women, particularly those with high viral load and positive HBeAg test have higher risk of passing the infection to their new-born during delivery [28]. Furthermore, it is known that high proportion of children infected at birth eventually evolve to chronicity, and birth dose is shown to reduce up to 90% of this risk [29, 30]. Screening and treating pregnant women as well as birth dose vaccine, are key to achieving the national target.

Very good agreement between CLEIA and the Alere Determine™ HBsAg rapid test was reported in this study and suggest that this rapid test could be advocated for screening hepatitis B infection in resource limited setting where good laboratory facilities are limited.

HBV DNA was detected in 65.0% of HBsAg positive samples with high viral load (> 5 log IU/ mL) estimated in 5 cases. Phylogenetic analysis reported 100% homology between case 18,025-BUR (child) and case 18,026-BUR (mother) and this imply the persistence of mother to child vertical transmission. The mother of the other child case (18311-BUR) was HBsAg negative, and therefore classified as horizontal transmission. Among 240 mother and child pairs in this study, one pair is evident to be infected through mother-to-child vertical transmission (0.42%) and the rest one is infected through horizontal transmission (0.42%) possibly from the household contacts. Sequence comparison in GenBank shows a new recombinant genotype and might have an implication in the clinical manifestation and treatment approaches. Here by this study, the rate of mother-to-child transmission of HBV infection showed very low percentage (< 1%) which indirectly proves the effectiveness of hepatitis B vaccination to all infant as national immunization program in Burkina Faso. The similar study in Cambodia also showed the effectiveness of hepatitis B vaccination including hepatitis B birth dose vaccine within 24 h after delivery which significantly reduce HBsAg prevalence to less than 1% in post-vaccine cohort of 5–7 years old children [31].

In January 2006, the government of Burkina Faso introduced hepatitis B vaccine as free immunization for children. However, among the 37.5% of children with protective level of HBsAb, 60.0% were secondary to past exposure to the wild type of the virus. The reasons behind this could be an inadequate timing of HBV vaccination in a region with high risk of infection, and the low quality of available doses due to multiples reasons including the challenges in maintaining adequate cold chain [32], sub-quality of vaccine dose available, individual variation in immune response or a poor vaccine coverage. The low response rate reported in the current study contrasts with results reported in others studies conducted in the country. Indeed, Kissou et al [33], and Ouedraogo et al [34], among under 3 years age children all reported approximately 90% response rates. The reasons behind these high rates could be related to the fact they did not discriminate participant immune due to vaccine or past infection, and also the timing between vaccine completion and their analyses. The study in Gambia documented that hepatitis B vaccine was 84% effective against infection and 94% effective against chronic carriage. But breakthrough infection and chronic carriage could occur more in vaccinated infants of mothers positive for hepatitis B surface and e antigens were than infants of uninfected mothers [35]. The study in Egypt also showed the existence of breakthrough infection despite of complete three dose hepatitis B vaccine with 57.2% of the sero protective rate after complete 3 doses hepatitis B vaccine [36]. In univariate analysis, serological vaccination rate was significantly low in children born at home than their peers born in health centres (OR = 0.4, 95% CI, 0.1–0.9, *p* = 0.048). Even though this was not significant in multivariate analysis, the trend was maintained (aOR = 0.4, 95% CI, 0.1–1.1, *p* = 0.073). This could be explained by the fact that mothers who delivered at home were less likely to visit health centres and then vaccinate their children. This hypothesis is supported by the significant low rate of well vaccinated children born at home compared to their counterpart born in health centres (*p* = 0.006). This stresses on the necessity to end home delivery in the rural area in order to improve vaccine effectiveness. This is in line with the WHO recommendation of promoting health centre delivery for all women. Awareness raising on the effects of home birth could help achieved this goal.

The prevalence of anti-HCV in this study was 5.4% in mothers and 2.1% among children. Meda et al. [16], found a similar prevalence 3.6%, Zeba et al. [37], 4.4% among blood donors in 2013. This is indicative of a stagnant prevalence of hepatitis C infection in the country. Indeed, HCV was long ignored as public health problem in the country and was therefore given little attention. With the availability of high effective DAAs in the country, more interventions should be initiated. Anti-HCV positivity was significantly higher in participant with history of blood transfusion; however risk analysis did not show significant association as described elsewhere in a Cambodian study [38]. The reasons behind this finding may include a few populations with history of transfusion in the current study. A larger sample size could provide more details regarding blood safety and risk factors, to guide future interventions.

## Conclusions

In this study, serological analysis suggested that the carriage rate of HBsAg is gradually decreasing in rural Burkina Faso and 2020 WHO African region of HBsAg prevalence under 2% target in children under 5 years age is already met. In risk analysis, factors including older age, being female and traditional scarification positively contributed to the occurrence of the infection and need to be considered in policy formulations. Moreover, residual humoral immunity rate was not satisfactory in the children group, and home birth was reported as the main contributing factor of poor vaccination coverage and immunity response. Full genome analysis suggests that mother-to-child transmission represent is persistent, highlighting the importance of preventive measures in pregnant women including screening and treatment as well as the administration of HBV birth dose in their new-born babies at delivery. Furthermore, HBV genotype E was predominant, followed by genotype A and a new recombinant genotype A3/E was reported for the first time in the country, and might modify the course of the infection and also jeopardize the effectiveness of current measures available in the country.

## Supplementary information

**Additional file 1.** Questionnaires used in the study. Appendix 1a is questionnaire for children and appendix 1b for mothers. These questionnaires were solely developed for this study and it mainly included the demographic information, vaccination status of the children, general knowledge on the hepatitis B virus infection and the available of vaccination.

**Additional file 2.** Serological profile among study participants in Nanoro health district area in 2018, Burkina Faso. This table describes the positive rates of HBV and HCV seromarkers classified by its infection status.

**Additional file 3.** Hepatitis B surface antigen (HBsAg)-positive cases among mothers and their children in Nanoro, Burkina Faso, 2018. This table describes the complete information of the HBV strains detected from HBsAg positive mothers and their children, including seromarkers status, viral load, the genotypes and the length of sequences.

## Data Availability

The dataset used and analysed during the current study is available from the corresponding author on reasonable request. All sequenced data of Burkina Faso HBV isolates are registered at GenBank via DDBJ. The sequences can be accessed at http://getentry.ddbj.nig.ac.jp/top-e.html with accession number of LC513651-LC513657 for HBV and LC513766 for HCV. If any trouble is had accessing the data, the sequences are available from the corresponding author upon reasonable request.

## Abbreviations

95%CI: 95% exact confidence intervals
CLEIA: Chemiluminescent enzyme immunoassay
COI: Cut-off index
CRUN: Clinical research unit of nanoro
DAA: Direct antiviral agents
DBS: Dried blood spots
HBsAg: Hepatitis b surface antigen
HBV: Hepatitis b
HCV: Hepatitis c
HCVAb: Hcv antibody
HDSS: Health and demographic surveillance system area
NAT: Nucleic acid amplification test
OR: Odd-ratios
RNG: Random number generator
TBS: Tris-buffered saline
