# Supplementary material for: The changing epidemiology of hepatitis B and C infections in Nanoro, rural Burkina Faso: a random sampling survey
Source: BMC Infect Dis. 2020 Jan 15;20:46. doi: 10.1186/s12879-019-4731-7 (PMC6964067; doi:10.1186/s12879-019-4731-7)
Supplement: Supplementary file 1 — Additional file 1. Questionnaires used in the study. Appendix 1a is questionnaire for children and appendix 1b for mothers. These questionnaires were solely developed for this study and it mainly included the demographic information, vaccination status of the children, general knowledge on the hepatitis B virus infection and the available of vaccination. [file 12879_2019_4731_MOESM1_ESM.zip › 19 12 05 Appendix 1aR5.docx]

| **Appendix 1a: Questionnaire for children**  **INSTITUT DE RECHERCHE**  **EN SCIENCES DE LA SANTE** |  |  |
| --- | --- | --- |
| **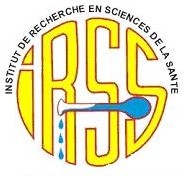** |  |  |

**Direction Régionale**

**du Centre Ouest**

Unité de Recherche Clinique de Nanoro

**SURVEY ON VIRAL HEPATITTIS INFECTION AMONG CHILDEN AND MOTHER IN NANORO, BURKINA FASO**

1

Code Village

# Paire

**ID unique # :**

**HDSS household number: _________________________**

**Date of interview: ……..…./…………/…………**

Does the parent sign the inform consent form? Yes . No

If no, do not include in the study;

If yes, provide date of consent signature: **|**__|__| |__|__| |__|__|__|__|.

*dd mm yyyy*

**Conduct of the survey**:

| **No.** | **Questions et filters** | | | **Response and coding** | **Instructions** | |
| --- | --- | --- | --- | --- | --- | --- |
| **Q01** | Child first name and name | | | _________________________________________ | In capital letters | |
| **Q02** | First name and name of the parent or legal guardian | | | _________________________________________ | In capital letters | |
| **Q03** | Relation with child | | | _________________________________________ | In capital letters | |
| **Q04** | Village de residency | | | __________________________________________ | In capitals |  |
| **Q05** | | Ethnic group | | Mossi……………………………………………….. 1  Gourounsi………………………………………… 2  Peuhl……………………………………………….. 3  Other, detail………………………………….. 4 | circle one answer | |
| **Q06** | | Occupation | | Preschool age child…………………………………. 1  kindergarten………………………………………… 2  Primary……………………………………………. 3  Secondary………………………………………… 4  Unschooled…………………………………….. 5 | Cercle one answer | |
| **Q07** | | Date of birth? | | /___/___/ /___/___/ /___/___/___/___/  dd *mm yyyy*  If unknown, give : /___/___/ (years) | Born between 2007 and 2017 |  |
| **Q08** | | Sex? | | Male ………………………………………………… 1  Female………………………………………….……….. 2 | circle one answer |  |
| **Q09** | | Place of birth? | | Public peripheral hospital 1  reference hospital 2  Private hospital 3  Home birth 4  Others (detail) 5  Unknown 6 | circle one answer |  |
| **Q10** | | Person Who did the delivery | | Health professional 1  village member 2  Family member 3  Other (detail)  4  Unknown 5 | circle one answer |  |
| **Q11** | | | Vaccination record health booklet available? | Yes …………………………………………………………….1  No……………………………………………………………2 | circle one answer |  |
| **Q12** | | | Vaccine received by child | 1. HepB 0 (dose de naissance)  Date……………………  2. BCG + VPO 0 Date……………………  3. Penta+VPO+Pneumo+Rota - 1  Date……………………  4. Penta+VPO+Pneumo+Rota - 2 Date……………………  5. Penta+VPO+Pneumo+Rota - 3 Date……………………  6. RR 1+ VAA Date……………………  7. RR2 Date……………………  8. Other vaccines (detail below)  Vaccine 1 ___________________ Date……………………  Vaccine 2____________________ Date……………………  Vaccine 3 ____________________ Date……………………  9. no vaccine received | circle all applicable answers.  *Penta= DTC-HepB-Hib |  |
| **Q13** | | | Hepatitis B vaccine birth dose? | Yes 1  No 2  Unknown 3 | Circle one answer |  |
| **Q14** | | | If yes, how long after birth? | Within the 24 hours after birth 1  Between 24 and 48 hours after birth. 2  Unknown 3 | Circle one answer |  |
| **Q15** | | | Any pentavalent vaccine dose received?  *(given at 2, 3, and 4 months after birth)* | Yes 1  No 2  Unknown 3 | Circle one answer |  |
| **Q16** | | If no Penta administered, give reason | | Homebirth, No vaccine available 1  Health center far……………………. 2  Don’t know, it was necessary ……………….3  Don’t trust vaccines……………… 4  Vaccine have side effects……………. 5  High cost of vaccine……….……………………….6  Other (detail) ……………………………………………7  No answer……………………………………………….. 8 | Circle all applicable answers |  |
| **Q17** | | In which health center the child received majority of his vaccination?  ____________________ | | Peripheral health facility 1  Public hospital 2  Private center 3  other, detail 4  Unknown 5  never vaccinated 6 | Cercle one answer |  |
| **Q18** | | How do you get to the health facility for you child vaccination? | | by walk 1  by bike 2  by motorbike 3  by car 4  other, detail 5 | Circle one answer |  |
| **Q19** | | How long does it take from your home to the health center | | ___________ minutes | digits |  |
| **Q20** | | History of piercing? | | Yes 1  No 2  Unknown 3  No answer…………………………………………4 | circle one answer |  |
| **Q21** | | History of surgery | | Yes 1  No 2  Unknown 3  No answer…………………………………………4 | Cercle one answer |  |
| **Q22** | | History of blood transfusion? | | Yes 1  No 2  Unknown 3  No answer…………………………………………4 | Cercle one answer |  |
| **Q23** | | History of tattoo? | | Yes 1  No 2  Unknown 3  No answer…………………………………………4 | Circle one answer |  |
| **Q24** | | History of scarification | | Yes 1  No 2  Unknown 3  No answer…………………………………………4 | Circle one answer |  |
| **Q25** | | Genital mutilation for girls | | Yes 1  No 2  Unknown 3  No answer…………………………………………4 | Circle one answer |  |
| **Q26** | | History of circumcision for male infants | | Yes 1  No 2  Unknown 3  No answer…………………………………………4 | Cercle on answer |  |
| **Q27** | | If yes to Q26, status of the person who perform | | Heath profession 1  Family member 2  Other, detail  3  Unknown 4  No answer…………………………………………5 | Circle one response |  |

**Blood sample**

| **Q28** | RDT perform? | Positive 1  Negative 2  Invalid 3 | Circle one response |
| --- | --- | --- | --- |
| **Q29** | HemaSpot dried blood spot collected | Yes 1  No 2  If no, give reason: …………………………………………  …………………………………………………………………. | Circle one response |

**Code of agent.......………………… Signature:..........................**
