# Supplementary material for: The changing epidemiology of hepatitis B and C infections in Nanoro, rural Burkina Faso: a random sampling survey
Source: BMC Infect Dis. 2020 Jan 15;20:46. doi: 10.1186/s12879-019-4731-7 (PMC6964067; doi:10.1186/s12879-019-4731-7)
Supplement: Supplementary file 1 — Additional file 1. Questionnaires used in the study. Appendix 1a is questionnaire for children and appendix 1b for mothers. These questionnaires were solely developed for this study and it mainly included the demographic information, vaccination status of the children, general knowledge on the hepatitis B virus infection and the available of vaccination. [file 12879_2019_4731_MOESM1_ESM.zip › 19 12 05 Appendix 1bR5.docx]

| ***Appendix 1b: Questionnaire for Mother***  **INSTITUT DE RECHERCHE**  **EN SCIENCES DE LA SANTE** |  |  |
| --- | --- | --- |
| **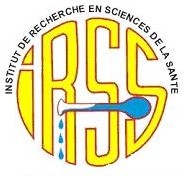** |  |  |

**Direction Régionale**

**du Centre Ouest**

Unité de Recherche Clinique de Nanoro

**SURVEY ON VIRAL HEPATITTIS INFECTION AMONG CHILDEN AND MOTHER IN NANORO, BURKINA FASO**

Code village

# Paire

2

**ID unique # :**

**HDSS household number: _________________________**

**Date of interview: ……..…./…………/…………**

Does the participant provide a written inform consent? yes . No

If no, not included in the study;

If yes, provide date of signature: **|**__|__| |__|__| |__|__|__|__|.

dd *mm yyyy*

**Conduct of the survey**:

| **N^o^** | **Questions and filters** | **Responses and code** | **Instructions** |
| --- | --- | --- | --- |
| **Q30** | First name and name | __________________________________________ | In capital letters |
| **Q31** | village of residency | __________________________________________ | In capital letters |
| **Q32** | Ethnic group | Mossi…………………………………………………………… 1  Gourounsi…………………………………………………… 2  Peuhl…………………………………………………………... 3  Other, detail……………………………………………..… 4 | circle one answer |
| **Q33** | Occupation | Farmer……..……………………………………………. 1  Civil servant……………………………………………….. 2  Merchant ……………………………………………… 3  Factory employee ………………………………………… 4  Military/paramilitary………………………………….. 5  Other, detail………...……………..……………………... 6  No answer………...………………………………….. 7 | circle one answer |
| **Q34** | Partner occupation | farmer……………………………………………………. 1  civil servant…………………………………………….. 2  Merchant ……………………………………………… 3  factory employee ………………………………………… 4  Military/paramilitary………………………………….. 5  Other, detail………...……………..……………………... 6  No answer………...………………………………….. 7 | circle one answer |
| **Q35** | Level of instruction? | Never been at school…………………………………..1  Primary…………………………………..………….. 2  Secondary (6 – 3^rd^ grade)………………….…… 3  Secondary (2^nde^- terminal grade)……………..... 4  University level…………………….……………….… 5  Non-formal education………………………. 6  no answer……………………………………. 7 | circle one answer |
| **Q36** | Your birth date? | /___/___/ /___/___/ /___/___/___/___/  *dd mm yyyy*  If unknown , give age: /___/__,_/ (years) | write date of birth |
| **Q37** | Marital status | Married…………………………………..……………. 1  Single…………………………………………… 2  Divorced…………………………………….……….. 3  widowed…………………………………………….……. 4  other, detail………...……………..…………… 5  no answer…………………………………….. 6 | Circle one answer |
| **Q38** | Marital status of the partner ? | Monogamous 1  Polygamous 2  other details………...……………..……………….3  Unknown 4 | circle one answer |
| **Q39** | Number of children of your partner | Same mother .............. /___/___/  Differentmother ........ /___/___/ | give number |
| **Q40** | Are you pregnant? | Yes 1  No 2  Unknown 3  No answer 4 | Circle one answer |
| **Q41** | Birth place? | Peripheral health center 1  Hospital 2  Private center 3  Home 4  Other, detail………...……………..………………….5  Unknown 6 | Circle one answer |
| **Q42** | Birth person | Health professional 1  Village assistant 2  Family member 3  Other, specify  5  Unknown 6 | Circle one answer |
| **Q43** | Do have health booklet ? | Yes 1  No 2 | Circle one answer |
| **Q44** | Head about hepatitis B ? | Yes 1  No 2  Unknow 3  No answer 4 | Circle one answer |
| **Q45** | Know about mother to child transmission of hepatitis B? | Ye 1  No 2  Unknown 3  No answer 4 | Circle one answer |
| **Q46** | Knowledge of sexual transmission? | Yes 1  No 2  Unknown 3  No answer 4 | Circle one answer |
| **Q47** | Known about transmission through contaminated material? | Yes 1  No 2  Unknown 3  No answer 4 | Circle one answer |
| **Q48** | Knowledge whether there is prevention method? | Yes 1  No 2  Unknown 3  No answer 4 | Circle one answer |
| **Q49** | Ever been vaccinated against HBV? | yes, when I was child 1  Yes, when I was adult 2  No, I have hepatitis infection markers positive 3  No 4  Unknown 5 | Circle one answer |
| **Q50** | If yes, How many doses ? | 1 dose…………………………………………………….. 1  2 doses…………………………………………………… 2  3 doses…………………………………………………… 3  4 doses et plus……………………………………….. 4 | Circle one answer |
| **Q51** | Date of doses | 1. First Date…………………… 2. Second Date…………………… 3. Third Date…………………… 4. Fourth Date…………………… | Circle all applicable date and write down the date |
| **Q52** | If no vaccination, give reason | Homebirth, No vaccine available 1  Health center far……………………. 2  Don’t know, i was necessary ……………….3  Don’t trust in vaccine……………… 4  Vaccine have side effects……………. 5  High cost of vaccine……….……………………….6  Other (detail) ……………………………………………7  No answer……………………………………………….. 8 | circle all applicable responses |
| **Q53** | Health center for vaccination  ___________________ | Peripheral health facility 1  Public hospital 2  Private center 3  other, detail 4  Unknown 5  never vaccinated 6 | Circle one answer |
| **Q54** | How do you get to the health facility for you child vaccination? | by walk 1  by bike 2  by motorbike 3  by car 4  other, detail 5 | Circle one answer |
| **Q55** | How long does it take from your home to the health center | ___________ minutes | digit |
| **Q56** | History of piercing | Yes 1  No 2  Unknown 3  No answer…………………………………………4 | circle one answer |
| **Q57** | History of surgery? | Yes 1  No 2  Unknown 3  N answer…………………………………………4 | Circle one answer |
| **Q58** | History of blood transfusion | Yes 1  No 2  Unknown 3  No answer…………………………………………4 | Circle one answer |
| **Q59** | Tattoo? | Yes 1  No 2  Unknown 3  No answer …………………………………………4 | Circle one answer |
| **Q60** | history of scarification | Yes 1  No 2  Unknown 3  No answer…………………………………………4 | Circle one answer |
| **Q61** | Genital mutilation? | Yes 1  No 2  Unknown 3  No answer…………………………………………4 | Circle one answer |

**PRELEVEMENT DES ECHANTILLONS**

| **Q62** | TDR | Positive 1  Negative  Invalid 3 | Circle one answer |
| --- | --- | --- | --- |
| **Q63** | Dried blood spot | Yes 1  No 2  If no, reason: …………………………………………  …………………………………………………………………. | Circle one answer |

**Code of agent.......………………… Signature:..........................**
