# Supplementary material for: The changing epidemiology of hepatitis B and C infections in Nanoro, rural Burkina Faso: a random sampling survey
Source: BMC Infect Dis. 2020 Jan 15;20:46. doi: 10.1186/s12879-019-4731-7 (PMC6964067; doi:10.1186/s12879-019-4731-7)
Supplement: Supplementary file 2 — Additional file 2. Serological profile among study participants in Nanoro health district area in 2018, Burkina Faso. This table describes the positive rates of HBV and HCV seromarkers classified by its infection status. [file 12879_2019_4731_MOESM2_ESM.docx]

**Appendix 2**. Serological profile among study participants in Nanoro health district area in 2018, Burkina Faso

|  |  | **Mothers (n =240)** | | **Children (n =240)** | |
| --- | --- | --- | --- | --- | --- |
| **Categories of subjects** | **Criteria** | **N (+)** | **p (95% CI)** | **N (+)** | **p (95% CI)** |
| Susceptible | HBsAg (-) HBsAb (-) & HBcAb (-) | 26 | 10.8 (7.5-15.5) | 62 | 25.8 (20.7-79.3) |
| Immune due to past infection | HBsAb (+) &HBcAb (+) | 14 | 5.8 (3.5-9.6) | 54 | 22.5 (17.6-28.3) |
| Immune due to vaccine | HBsAb (+) HBcAb (-) | 0 | 0.0 (0.0-1.5) | 36 | 15.0 (11.0-20.1) |
| Acute or chronic infection | HBsAg (+) HBsAb (+) or HBcAb (+) | 15 | 6.3 (3.8-10.1) | 2 | 0.8 (0.2-3.3) |
| Exposure to HBV anytime | HBcAb (+) or HBsAg | 214 | 89.2 (84.5-92.5) | 142 | 59.2 (52.8-65.3) |
| HCV infection | Anti-HCV (+) | 13 | 5.4 (3.2-9.1) | 5 | 2.1 (0.9-4.9) |
| HCV & HBV coinfection | HBsAg (+) & anti-HCV (+) | 0 | 0.0 (0.0-1.5) | 0 | 0.0 (0.0-1.5) |
