# Supplementary material for: The changing epidemiology of hepatitis B and C infections in Nanoro, rural Burkina Faso: a random sampling survey
Source: BMC Infect Dis. 2020 Jan 15;20:46. doi: 10.1186/s12879-019-4731-7 (PMC6964067; doi:10.1186/s12879-019-4731-7)
Supplement: Supplementary file 3 — Additional file 3. Hepatitis B surface antigen (HBsAg)-positive cases among mothers and their children in Nanoro, Burkina Faso, 2018. This table describes the complete information of the HBV strains detected from HBsAg positive mothers and their children, including seromarkers status, viral load, the genotypes and the length of sequences. [file 12879_2019_4731_MOESM3_ESM.docx]

**Appendix 3**: Hepatitis B surface antigen (HBsAg)-positive cases among mothers and their children in Nanoro, Burkina Faso, 2018

|  | **Isolate ID** | **Category** | **HBsAg** | **HBeAg** | | **HBeAb** |  | **HBcAb** |  | **DNA quantification**  **(copies/mL)** | **HBV PCR (Nested)** | | **Genotype** | **Full sequence**  **length(bp)** |
| --- | --- | --- | --- | --- | --- | --- | --- | --- | --- | --- | --- | --- | --- | --- |
|  |  |  |  |  |  |  |  |  |  |  | **PCR-WA†** | **PCR- S‡** |  |  |
| 1 | 18002-BUR | Mother | 5.8 | 0.1 | - | 100 | + | 185.8 | + | ND | - | + | E | NS |
| 2 | 18025-BUR | Child | 2000 | 101.4 | + | 8.3 | - | 4.4 | + | 3.2E+07 | + | NT | E | 3,212 |
| 3 | 18026-BUR | Mother | 2000 | 154.4 | + | 4.9 | - | 2.4 | + | 5.3E+07 | + | NT | E | NS |
| 4 | 18040-BUR | Mother | 212.9 | 0.1 | - | 100 | + | 164.8 | + | ND | - | + | A | NS |
| 5 | 18062-BUR | Mother | 1837.3 | 0.2 | - | 51.5 | + | 141.4 | + | ND | - | - | ND | NS |
| 6 | 18142-BUR | Mother | 2000 | 94.7 | + | 9.2 | - | 15.7 | + | 6.1E+07 | + | NT | A3/E | 3,215 |
| 7 | 18200-BUR | Mother | 1852.6 | 0.1 | - | 38.7 | - | 3.5 | + | 3.6E+02 | + | NT | E | 3,212 |
| 8 | 18306-BUR | Mother | 2000 | 6.5 | + | 40.6 | - | 89.7 | + | 5.8E+06 | + | NT | E | NS |
| 9 | 18311-BUR | Child | 2000 | 825.3 | + | 0.1 | - | 189.3 | + | 1.6E+07 | + | NT | A3/E | 3,215 |
| 10 | 18342-BUR | Mother | 3.4 | 0.1 | - | 72.3 | + | 69.5 | + | 5.3E+00 | - | - | ND | NS |
| 11 | 18354-BUR | Mother | 1412.8 | 0.1 | - | 52.1 | + | 55.4 | + | ND | - | + | A | NS |
| 12 | 18362-BUR | Mother | 1138.8 | 0.6 | - | 39.9 | - | 33.8 | + | 1.7E+04 | + | NT | E | NS |
| 13 | 18378-BUR | Mother | 1670.5 | 0.1 | - | 62.8 | + | 51.5 | + | 1.0E+03 | + | NT | E | NS |
| 14 | 18446-BUR | Mother | 881.1 | 0.1 | - | 42.7 | - |  | + | 8.5E+00 | - | - | ND | NS |
| 15 | 18456-BUR | Mother | 1321.6 | 0.1 | - | 91.7 | + | 54.7 | + | 6.1E+01 | + | NT | E | 3,212 |
| 16 | 18466-BUR | Mother | 549.7 | 0.1 | - | 100 | + | 90.9 | + | ND | - | - | ND | NS |
| 17 | 18472-BUR | Mother | 247.1 | 0.1 | - | 95.1 | + | 28.2 | + | ND | - | + | A | NS |

ND, not detected; NT, not tested; NS, testing not successful

**†** Long PCR (about 3 Kilo base pairs)

**‡** High sensitivity PCR (193 base pairs)
